# Extradigital Glomangioma of the Cutaneous Chest Wall

**DOI:** 10.7759/cureus.17910

**Published:** 2021-09-12

**Authors:** Haidar N Alyaseen, Hussain A Al Ghadeer, Mukhtar E Al-Ghanim, Hameed H Aljawad, Carlos R Cordoba

**Affiliations:** 1 General Surgery, Almoosa Specialist Hospital, Al-Ahsa, SAU; 2 Paediatrics, Maternity and Children Hospital, Al-Ahsa, SAU; 3 General Surgery, King Faisal University, Al-Ahsa, SAU; 4 Pathology, Almoosa Specialist Hospital, Al-Ahsa, SAU; 5 Plastic Surgery, Montreal University Hospital Centre, Montreal, CAN

**Keywords:** glomus tumor, glomangioma, atypical location, chest wall, extradigital

## Abstract

Glomus tumors (GTs) are rare benign tumors as a result of hyperplasia of glomus body. GT most commonly involves the subungual areas and rarely involves extra-digital sites. The clinical presentation of a glomus tumor is a triad of symptoms consisting of pain, cold intolerance, and pinpoint tenderness. Even though glomus tumors are benign, they can infrequently be malignant. Despite their benign nature, these lesions can cause disabling symptoms, therefore proper diagnosis and treatment is important. In this report, we present a 35-year-old Saudi male with a painful lesion on the right side of the chest wall at the posterior axillary line for seven years, with recent progressive growth and symptoms. Diagnosis of extra-digital glomangioma of the chest wall in this patient was confirmed by histopathology. The patient was managed by complete surgical excision of the lesion with the resolution of pain and without recurrence.

## Introduction

Glomus tumors (GTs) are benign neoplastic lesions arising from proliferating neuromyoarterial structures. These structures are also called glomus bodies. The glomus bodies are found in the stratum reticularis of the dermis throughout the body. They participate in thermoregulation and control blood pressure by changing blood flow in the skin [1]. They represent less than 2% of all soft tissue tumors [2]. The majority of the cases are located digitally at subungual sites in the fingers and toes, where glomus bodies are in abundance. Therefore, extradigital GTs are rare. They are characterized by a clinical triad of severe pain, localized tenderness, and cold temperature sensitivity. Extradigital GT is usually asymptomatic and smaller in size when compared to digital GT, making the diagnosis challenging [3]. GTs are classified histologically into solid tumors, glomangiomas, and glomangiomyomas based on the predominant component [4]. GTs rarely become malignant [5].

However, 60% of extradigital GTs cases are seen on the upper extremity, and only 24% are seen on the trunk area [6]. To the best of our knowledge, only eight cases of extradigital GTs involved chest wall are reported in the literature. In this report, we are presenting a 35-year-old Saudi male presented with a progressively painful lesion of the chest wall. Histopathology confirmed the diagnosis of a GT in the form of a glomangioma.

## Case presentation

**Figure 1:**
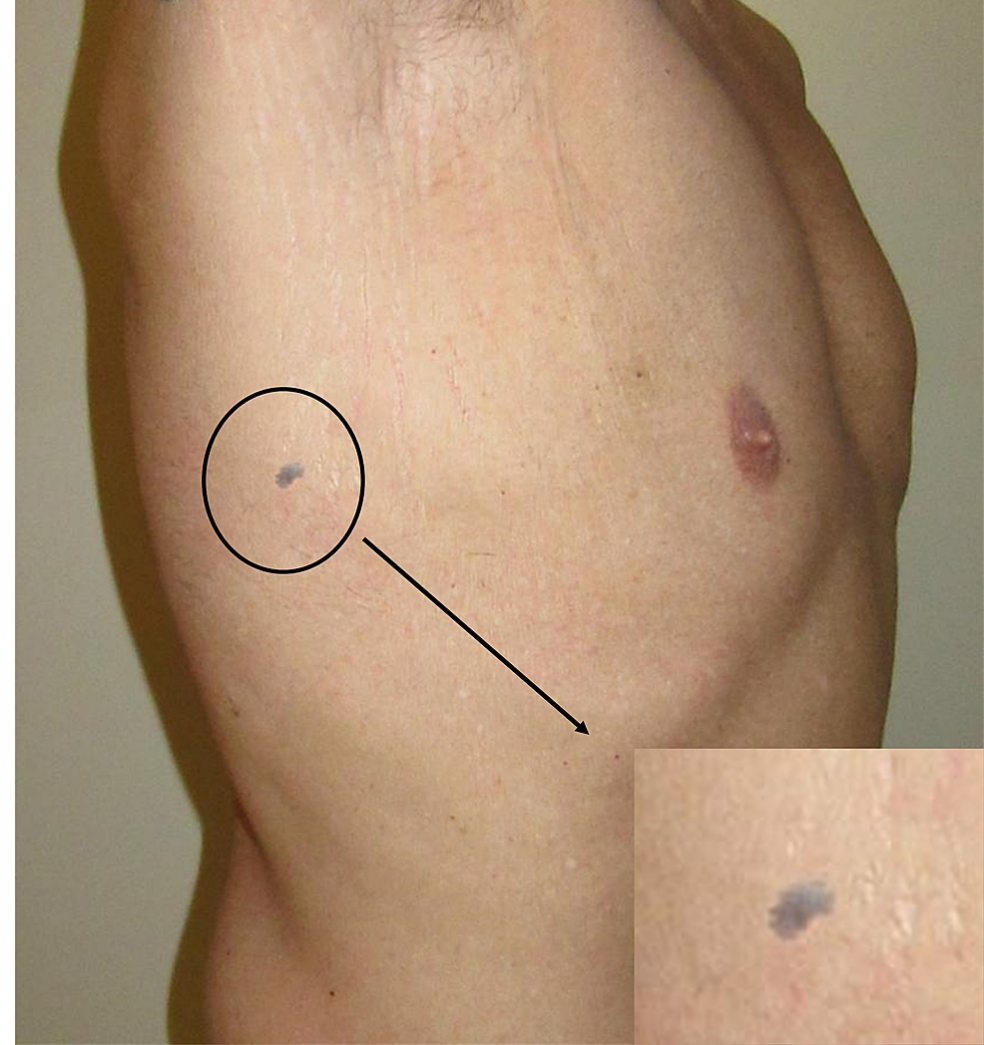
A single small well-circumscribed bluish papule over the right side of chest wall.

We present the case of a 35-year-old Saudi male engineer who has no past medical or surgical history. He was referred by dermatology to our plastic surgery clinic at Almoosa Specialist Hospital, Al-Ahsa, Saudi Arabia. The patient presented with an atraumatic painful single small well-circumscribed bluish papule over the right side of the chest wall along the posterior axillary line, for the past seven years (Figure 1). He complained of unusual tingling and burning sensation at the lesion. The lesion became progressively painful, and the patient gave a history of a seasonal variation of symptoms, being more painful with exposure to cold. This prompted him to seek medical attention. The papule measured 1 cm in diameter. It was tender only on palpation. The lesion was firm in consistency and noncompressible. He denied any family history. The physical exam was otherwise unremarkable. At another hospital, the patient had undergone routine hematological investigations that were within normal range and radiological imaging without achieving a definite diagnosis. We excised the lesion in Toto under local anesthesia. The patient went on to heal without any complications and with complete resolution of his pinpoint tenderness on palpation.

**Figure 2:**
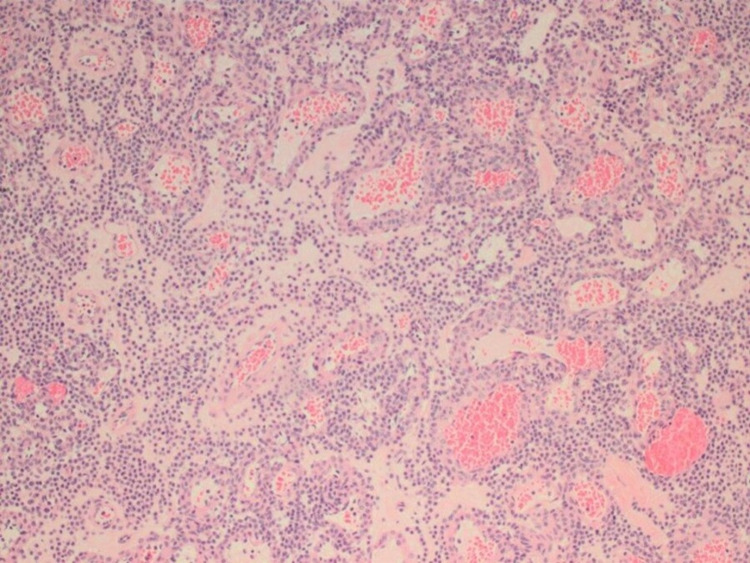
A section of this lesion composed of uniform round-shaped cells with indistinct cells and rounded sharply pinched out nucleus with amphophilic to eosinophilic cytoplasm. The nuclei of the cell are centrally located and have homogenous chromatin and inconspicuous nucleoli, no mitoses seen. The background of the lesion shows a prominent vascular component. Histopathology finding: glomangioma.

**Figure 3:**
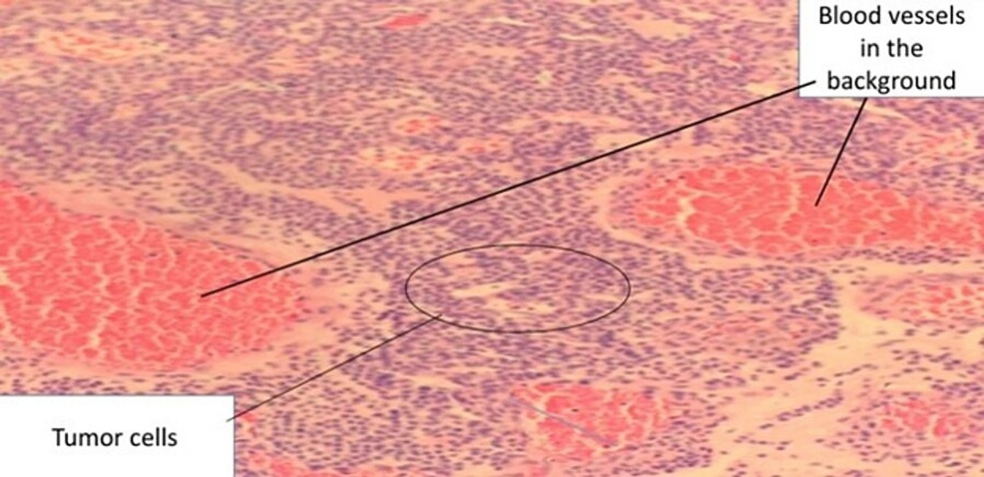
Tumor cells in the background of blood vessels.

**Figure 4:**
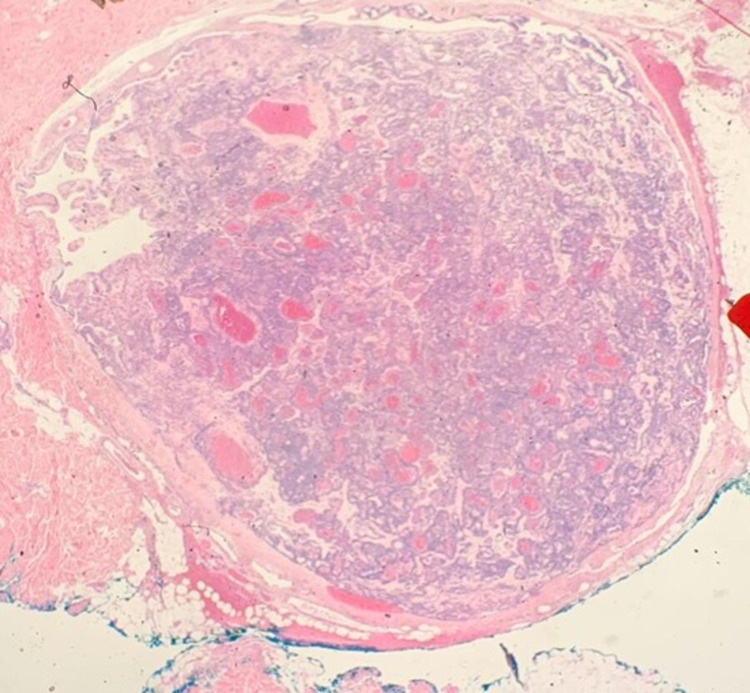
Section of skin and subcutaneous tissue containing well-delimited lesion of vascular nature based at the reticular dermis and dermal subcutaneous junction.

Gross histopathological analysis revealed a single small well-circumscribed bluish papule originating from the right-sided chest wall. Microscopic examination showed a circumscribed mass with an abundance of vasculature components consisting of dilated capillaries that are lined by endothelial cells surrounded by multiple layers of glomus cells (Figures 2-4). A glomangioma diagnosis was made.

## Discussion

Glomus bodies also called neuromyoarterial structures are a specialized form of arteriovenous anastomosis that are predominantly found within the reticular dermis. These bodies are responsible for the regulation of the temperature and blood pressure through peripheral blood flow. The tumor develops as a result of hyperplasia of the glomus body [1]. In 1812, Wood first discovered a glomus tumor as a painful subcutaneous nodule that was cured by surgical excision [7]. Overall GTs represent less than 2% of all soft tissue tumors [2]. Therefore, most of the studies were reported as case reports due to their rarity.

Although the GTs are benign, malignancies were reported in few cases that presented as extradigital GT like in the esophagus [8], thyroid [9], heart [10], lung [11], and intestine [12]. Moreover, GTs can be lethal [11,13]. These malignant GTs highlight that an extradigital GT is at higher risk for developing malignancy. Glomus tumors are of benign pathology, however, there is a 1% risk of malignancy. There are some reported cases of glomus tumors that have metastasized. Some of the features that increase the likelihood of malignancy are: tumor size greater than 2 cm, tumor depth within tissues, an increase in abnormalities of mitotic figures, a mitotic rate more than 5 per high power field and a moderate to high nuclear grade. Meeting all of these criteria, the malignancy risk has been found to be up to 25% [14].

GTs occur frequently in the subungual area where glomus bodies are prevalent. Around 25% occurred in an unusual location making diagnosis difficult. GTs of the subungual area are predominantly seen in females. Other sites are not associated with gender but predominantly affect adults aged between 40 and 70 years [15,16]. Extradigital chest wall lesions reported in the literature are seen to occur predominantly in males in the fourth to sixth decade of life.

They can be found as a single or multiple lesions. Usually, multiple lesions are inherited as autosomal dominant in the globulin gene on chromosome 1p21-22 [17]. Furthermore, some studies implicated trauma as a predisposing factor for developing a GT, stating that glomus bodies proliferate in response to trauma [18,19]. Schiefer et al. reported 20 to 30% of the patients diagnosed with extradigital GT had a history of trauma during a 20-year study experience [1].

The classical clinical triad presentation is pain, with pinpoint tenderness localization, and cold hypersensitivity. Examination reveals a positive Love test (localized pain with pressure applied to the site of the lesion by a pin) and a Hildreth test (relief of pain and tenderness while a cuff is inflated around the affected limb) [20]. Extradigital glomus tumors tend to present in unusual locations, with atypical presentations leading to misdiagnosis and chronic pain. A glomangioma is the most common histological subtype of extradigital tumors. When a patient presents with localized extradigital pain or an asymptomatic subcutaneous red or purple nodule, a glomus tumor should be considered as a differential diagnosis to avoid delay or misdiagnosis [6]. Although in the current report the patient was diagnosed with extradigital GT, he presented with classical manifestations but there was a delay of the diagnosis due to its unusual location. This emphasizes that GTs should be considered in the differential diagnosis.

The diagnosis of GT can be established by imaging studies and confirmed by histopathology. Ultrasound (US) was initially used for locating the lesion and measuring its size. Doppler US is used for assessing the blood flow surrounding the lesion. Magnetic resonance imaging (MRI) has a high sensitivity of 90%, specificity of 50% with 97% positive predictive value, and a low negative predictive value of 20%. Even though this demonstrates that a negative MRI does not rule out a GT, it is useful in other differential diagnoses such as benign neural cell tumors like schwannomas, neuromas, and neurofibromas [21]. Extradigital GT in the literature showed similar findings on MRI [22].

GTs are classified histologically into three types based on the predominant component. The first type is solid, consisting of only a glomus body with poor vasculature and sparse smooth muscle. The second type is glomangioma with a prevalent vascular component. The third type is glomangiomyoma containing the glomus body, prevalent vascular and smooth muscle components [23]. Our patient was diagnosed with a glomangioma based on the histological finding that revealed a predominant vasculature component surrounding glomus bodies.

Complete surgical excision is the main treatment for a single lesion of GT with the resolution of symptoms [24]. A retrospective study of 110 patients with digital and 42 patients with extradigital glomus tumors, reported a cure rate of 90% with complete excision. However, in one-third of GT cases, recurrence was reported 2-3 years after surgical removal. This recurrence is most probably due to incomplete or improper removal of GT [6]. The recurrence rate reported in the literature ranged from 12 to 33% [1,3,25]. In the present study, the excised specimen submitted had tumor-free margins, and the patient did not experience any recurrence (Table 1).

**Table 1:** Reported cases of chest wall glomus tumor in the literature

Study	Gender	Age (years)	Duration of symptoms (years)	Location	Recurrence
Temiz et al. [26]	Not reported	Not reported	Not reported	Subcutaneous sternum	No
Kambhampati and Kambhampati [27]	Male	47	2	Subcutaneous left anterior chest wall	No
Neelaiah and Suryanarayanarao [28]	Male	46	2	Subcutaneous anterior chest wall	Not reported
Tsuruta et al. [29]	Female	19	5	Dorsal side of pectoralis major, Right anterior chest wall	No
Yim et al. [30]	Male	41	Not reported	Deep in the chest wall muscles, Right lateral chest wall	Yes
Uchiyama et al. [31]	Male	50	10	Right 3rd intercostal space	No
Schneller [32]	Male	30	10	Multifocal in intercostal spaces, Left posterior chest wall (largest one)	Not reported
Zanjani et al. [33]	Male	63	15	Left chest wall (in the mid-axillary line)	No
Our patient	Male	35	7	Right chest wall (posterior-axillary line)	No

## Conclusions

Extradigital GT may be challenging to diagnose due to their occasional atypical presentation and location. Therefore, it is essential to include the glomus tumor in the differential diagnosis of a patient presenting with an extradigital well-localized painful or asymptomatic subcutaneous lesion. Early diagnosis is important to avoid a delay in treatment causing chronic pain and impairing the quality of life. Complete excision is the effective and curative treatment and histopathologic analysis confirms the diagnosis.

## References

[1] Schiefer TK, Parker WL, Anakwenze OA, Amadio PC, Inwards CY, Spinner RJ (2006). Extradigital glomus tumors: a 20-year experience. Mayo Clin Proc.

[2] Hartert M, Wolf M, Marko C, Huertgen M (2019). Glomus tumor of the trachea - Synopsis of histology & methodology of treatment. Respir Med Case Rep.

[3] Santoshi JA, Kori VK, Khurana U (2019). Glomus tumor of the fingertips: a frequently missed diagnosis. J Family Med Prim Care.

[4] Venugopal PR (2015). Extradigital glomus tumor - a rare cause for undiagnosed chronic pain in unusual sites. Indian J Surg.

[5] Sacchetti F, De Gori M, Grossi S, Bonadio GA, Capanna R (2019). An exceptional case of malignant glomus tumor and a review of the literature. Acta Orthop Traumatol Turc.

[6] Lee DW, Yang JH, Chang S, Won CH, Lee MW, Choi JH, Moon KC (2011). Clinical and pathological characteristics of extradigital and digital glomus tumours: a retrospective comparative study. J Eur Acad Dermatol Venereol.

[7] Wood W (1812). On painful subcutaneous tubercle. Edinb Med Surg J.

[8] Bali GS, Hartman DJ, Haight JB, Gibson MK (2013). A rare case of malignant glomus tumor of the esophagus. Case Rep Oncol Med.

[9] Chung DH, Kim NR, Kim T, Ahn J, Lee S, Lee YD, Cho HY (2015). Malignant glomus tumor of the thyroid gland where is heretofore an unreported organ: a case report and literature review. Endocr Pathol.

[10] Balisan OP, Radin CPT II, Arias R, Templo F Jr (2018). Malignant glomus tumor of the heart in a 64-year-old male presenting with stroke. PJP.

[11] Wang S, Ding C, Tu J (2015). Malignant glomus tumor of the lung with multiple metastasis: a rare case report. World J Surg Oncol.

[12] Chen J-H, Lin L, Liu K-L (2020). Malignant glomus tumor of the intestinal ileum with multiorgan metastases: a case report and review of literature. World J Gastroenterol.

[13] Liu Y, Wu R, Yu T, Cao Y, Lu L (2019). Malignant glomus tumor of the thyroid gland: a case report. J Int Med Res.

[14] Muneer M, Alkhafaji A, El-Menyar A, Al-Hetmi T, Al-Basti H, Al-Thani H (2016). Intravascular extra-digital glomus tumor of the forearm. J Surg Case Rep.

[15] Folpe AL, Fanburg-Smith JC, Miettinen M, Weiss SW (2001). Atypical and malignant glomus tumors: analysis of 52 cases, with a proposal for the reclassification of glomus tumors. Am J Surg Pathol.

[16] Calandruccio J, Jobe M (2017). Tumors and tumorous conditions of the hand. In: Campbell’s Operative Orthopaedics.

[17] Puchala M, Kruczynski J, Szukalski J, Lianeri M (2008). Glomangioma as a rare cause of knee pain. A report of two cases. J Bone Joint Surg Am.

[18] Morey VM, Garg B, Kotwal PP (2016). Glomus tumours of the hand: review of literature. J Clin Orthop Trauma.

[19] Samaniego E, Crespo A, Sanz A (2009). Key diagnostic features and treatment of subungual glomus tumor. Actas Dermosifiliogr (English Edition).

[20] Giele H (2002). Hildreth’s test is a reliable clinical sign for the diagnosis of glomus tumours. J Hand Surg.

[21] Al-Qattan MM, Al-Namla A, Al-Thunayan A, Al-Subhi F, El-Shayeb AF (2005). Magnetic resonance imaging in the diagnosis of glomus tumours of the hand. J Hand Surg Br.

[22] Lee S, Le H, Munk P, Malfair D, Lee ChH, Clarkson P (2010). Glomus tumour in the forearm: a case report and review of MRI findings. JBR-BTR.

[23] Karuppiah SV, Knox D (2014). Elbow dislocation with complete triceps avulsion. Case Rep Orthop.

[24] Chun JS, Hong R, Kim JA (2014). Extradigital glomus tumor: a case report. Mol Clin Oncol.

[25] Kim SH, Suh HS, Choi JH, Sung KJ, Moon KC, Koh JK (2000). Glomus tumor: a clinical and histopathologic analysis of 17 cases. Ann Dermatol.

[26] Temiz G, Şirinoğlu H, Demirel H, Yesiloglu N, Sarici M, Filinte GT (2016). Extradigital glomus tumor revisited: painful subcutaneous nodules located in various parts of the body. Indian J Dermatol.

[27] Kambhampati SB, Kambhampati AP (2018). Subcutaneous glomus tumor of chest wall. IJCR.

[28] Neelaiah S, Suryanarayanarao VP (2005). Glomus tumour of the chest wall. Case Rep Clin Pract Rev.

[29] Tsuruta Y, Mori T, Yoshioka M (2003). A case of glomus tumor in chest wall. JACS.

[30] Yim IH, Will MB, Carnochan FM, Walker WS (2016). A glomus tumor with recurrence and malignant transformation in the chest wall: a cautionary tale of seeding?. Ann Thorac Surg.

[31] Uchiyama M, Kato T, Kunitani K, Kuwabara K (2011). Multiple glomus tumors in chest wall and buttocks (Article in Japanese). Kyobu Geka.

[32] Schneller J (2001). Multifocal glomangiomyomas in the chest wall of a young man. Arch Pathol Lab Med.

[33] Zanjani LO, Shafiee Nia B, Vosoughi F, Mirzaian E, Aghaghazvini L, Arabzadeh A (2021). An unusual case of chest wall glomus tumor presenting with axillary pain: a case report and literature review. Eur J Med Res.

